# Second Primary Cancer After Bladder Cancer: A Comprehensive Analysis of a National Cancer Registry

**DOI:** 10.1002/cam4.71427

**Published:** 2025-11-30

**Authors:** Tomas Buchler, Lucie Pehalova Kusova, Alina Pirshtuk, Jan Muzik, Marek Babjuk, Ladislav Dusek

**Affiliations:** ^1^ Department of Oncology, Second Faculty of Medicine Charles University and Motol University Hospital Prague Czech Republic; ^2^ Institute of Health Information and Statistics of the Czech Republic Prague Czech Republic; ^3^ Institute of Biostatistics and Analyses, Faculty of Medicine Masaryk University Brno Czech Republic; ^4^ Department of Urology, Second Faculty of Medicine Charles University and Motol University Hospital Prague Czech Republic

**Keywords:** bladder cancer, cancer registry, lung cancer, second primary cancer, survivorship

## Abstract

**Background:**

Bladder cancer (BC) is one of the most common cancers and many patients will experience long‐term survival. Non‐urothelial second primary cancers (SPC) relatively frequently occur in the population of BC survivors.

**Methods:**

Czech National Cancer Registry was the principal data source for this study. The risk of development of non‐urothelial SPC after BC was assessed by the standardized incidence ratio (SIR). The standardized mortality ratio (SMR) was utilized to determine the risk of death from SPC following BC.

**Results:**

Of the total of 61,314 patients with BC, a cohort of 12,645 survivors diagnosed with a SPC were analyzed. Compared to the general population, the SIR of neoplasia was significantly increased in BC survivors, with a SIR of 1.75 (95% confidence interval [CI] 1.72–1.78) for all cancers, especially during the first 5 years after BC diagnosis. The SMR was increased in BC survivors (1.25; 95% CI 1.21–1.29) for all cancers. Lung cancer frequently occurred as a SPC and was associated with high mortality in BC survivors. BC survivors also had a higher risk of laryngeal cancer and other cancers known to be strongly associated with smoking, but also of soft tissue tumors, colorectal cancer, chronic lymphocytic leukemia, and thyroid cancer.

**Conclusions:**

The present large registry‐based study shows that BC survivors have a significantly higher risk of being diagnosed with another primary cancer compared to the general population, especially during the first 5 years after BC diagnosis. In addition to smoking‐related cancers, increased incidence of some cancers not known to be associated with smoking was observed.

## 1 Introduction

Bladder cancer (BC) is one of the most common cancers worldwide, with more than 500,000 cases diagnosed annually [1]. Many patients diagnosed with bladder cancer will experience long‐term survival, especially those with non‐muscle invasive bladder cancer. Smoking, the major risk factor for bladder cancer is associated with an increased risk of numerous other malignancies, and second primary cancers (SPCs) are consequently common in bladder cancer survivors [2, 3, 4].

The understanding of the risk of specific SPCs is important for follow‐up planning and to improve risk management strategy for BC survivors. The risk and patterns of second primary cancers (SPCs) among survivors of BC and other urothelial cancers have been the subject of extensive research in recent years. Notably, studies from various countries and using diverse methodologies have consistently shown an increased risk of SPCs in this population [4, 5, 6, 7, 8, 9, 10, 11]. Nevertheless, there is currently a lack of evidence for cancer surveillance for BC survivors.

The objective of this retrospective registry‐based study was to evaluate the risk of metachronous non‐urothelial SPCs among BC survivors in the Czech Republic and to provide a basis for the optimal follow‐up of these patients that would include targeted symptom assessment and diagnostic approaches for detecting SPCs. For this analysis we obtained data on patients with BC from the Czech National Cancer Registry (CNCR).

## 2 Materials and Methods

### 2.1 Data Source

The database of the CNCR constituted the principal data source for this study. The CNCR database provides records of all neoplasms in the Czech population over more than 40 years (the analyzed period is 1977–2017) and constitutes an integral part of the complex oncological care. Registration of any malignancy is mandatory and anchored in legislation. For the more recent period, CNCR covers 100% of the Czech Republic. In total, it contains over 2.5 million individual records. BC cases were identified in accordance with the 10th Edition of the International Classification of Diseases (ICD‐10) based on codes C67.* for bladder cancer and D09.0 for bladder carcinoma in situ [12].

The inclusion criteria for the analysis were as follows: (1) BC diagnosis from 1977 to 2017; (2) another malignancy recorded in the CNCR database; (3) the second malignancy diagnosed later than 6 months after the diagnosis of BC (an arbitrarily chosen interval to exclude patients with synchronous second cancers). Patients with a malignant disease predating the diagnosis of BC were included if they developed another cancer later. For incidence and mortality calculations, we also utilized data on the demographic structure of the Czech Republic published by the Czech Statistical Office [13]. TNM stages have been used throughout the study, according to the TNM classification valid at the time of the diagnosis of BC or SPC case.

### 2.2 Statistical Analysis

Comparisons of baseline characteristics among BC patients, stratified by the presence of SPC, were summarized using counts and frequencies and tested with the Fisher exact test. For continuous characteristics, the Mann–Whitney test was employed. The risk of development of SPC after BC for individual locations was assessed by the standardized incidence ratio (SIR), comparing the observed and expected numbers of cases [14]. The standardized mortality ratio (SMR) was utilized to determine the risk of death from SPC following BC. Deaths attributed to BC were not included in SMR calculations. Person‐years at risk were calculated from the date of BC diagnosis to the patient's death from any cause or until the data cut‐off date (31 December 2017). Only metachronous tumors (i.e., those diagnosed more than 6 months after BC) were included in the SIR/SMR calculation. The 95% confidence interval (CI) for SIR/SMR was constructed upon the assumption of a Poisson distribution of observed values [15]. The age at BC diagnosis and SPC occurrence were described using the median and the 25th to 75th percentiles; again, only metachronous tumors were considered. The representation of clinical stages for selected SPC locations was described using counts and frequencies based on the time from the date of SPC diagnosis (within 5 years from BC, 5–10 years from BC, and more than 10 years from BC). The distribution of clinical stages of SPCs was compared to the reference distribution of first primary neoplasms of the corresponding site according to the CNCR database using Fisher's exact test.

## 3 Results

### 3.1 Baseline Characteristics of the Cohort

We obtained data from 61,314 patients diagnosed with primary malignant bladder tumors reported between 1977 and 2017, including 45,977 men (75%) and 15,377 women (25%). The cohort characteristics are shown in Table 1.

**TABLE 1:** Baseline characteristics of patients with bladder cancer with and without a second primary cancer.

	Patients with BC only (*N* = 49,633)	Patients with BC and SPC (*N* = 11,681)	*p*
Sex			
Male	36,675 (73.9%)	9302 (79.6%)	< 0.001^a^
Female	12,958 (26.1%)	2379 (20.4%)	
Age at diagnosis			
0–59 years	10,128 (20.4%)	2650 (22.7%)	< 0.001^a^
60–69 years	15,099 (30.4%)	4149 (35.5%)	
70–79 years	16,531 (33.3%)	3878 (33.2%)	
≥ 80 years	7875 (15.9%)	1004 (8.6%)	
Median age at diagnosis, years (5%–95% percentile)	69 (49–85)	67 (50–82)	< 0.001^b^
Clinical stage			
I	17,174 (34.6%)	5871 (50.3%)	< 0.001^a^
II	7408 (14.9%)	1886 (16.1%)	
III	2944 (5.9%)	456 (3.9%)	
IV	4511 (9.1%)	426 (3.6%)	
Not recorded	17,596 (35.5%)	3042 (26.0%)	
Year of BC diagnosis			
1977–1987	9638 (19.4%)	1170 (10.0%)	< 0.001^a^
1988–1997	11,501 (23.2%)	2730 (23.4%)	
1998–2007	14,007 (28.2%)	4558 (39.0%)	
2008–2017	14,487 (29.2%)	3223 (27.6%)	

Abbreviations: BC, bladder cancer; SPC, second primary cancer.

^a^ Fisher's exact test.

^b^ Mann–Whitney test.

There were differences between the population of patients with BC without a recorded SPC compared to patients with SPC. Males had a higher risk of developing a SPC compared to females.

A total of 14,699 cases of SPC occurred over the whole period of study (1977–2017); 86.2% of them (12,645 cases occurring in 11,681 patients) were diagnosed at least 6 months after the first cancer diagnosis of malignant bladder tumor, defining these cases as metachronous (Table S1). The most common non‐urothelial SPCs were non‐melanomatous skin cancer (3054 cases, 24.2% of all metachronous SPCs), lung cancer (1919 cases, 15.2%), prostate cancer in men (1374 cases, 10.9%), colorectal cancer (1237 cases, 9.8%), renal cancer (459 cases, 3.6%).

### 3.2 The Relative Risk of Second Primary Cancers Following Bladder Cancer

The SIR of neoplasia was significantly increased in BC survivors, with SIR of 1.75 (95% CI 1.72–1.78) for all cancers (without non‐melanoma skin cancers) and 1.49 (95% CI 1.39–1.62) for hematologic cancers.

**FIGURE 1:**
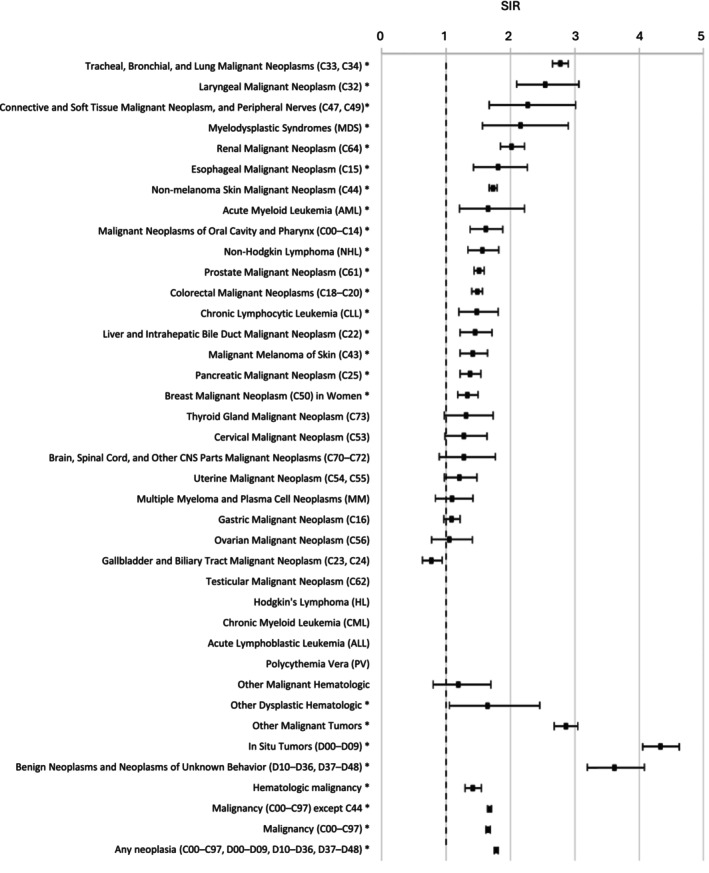
Risk of second primary cancer after bladder cancer.

As expected, cancers associated with smoking, including lung cancer and laryngeal cancer had the highest SIR (Table 2; Figure 1). Other smoking‐associated cancers with higher incidence compared to the general population included renal cell cancer, esophageal cancer, malignancies of the oral cavity and pharynx, myeloid malignancies, pancreatic cancer, and possibly colorectal cancer. Both men and women had an increased risk of lung cancer, with the SIR of 1.71 (95% IS 1.63–1.80) in men and 3.47 (95% IS 3.06–3.93) in women.

**TABLE 2:** Risk of metachronous second primary cancer after bladder cancer (*N* = 12,645).

Second primary neoplasm	Men		Women		All	
	*N*	SIR (95% IS)	*N*	SIR (95% IS)	*N*	SIR (95% IS)
Malignant neoplasms of oral cavity and pharynx (C00–C14)	139	1.14 (0.96–1.34)	24	1.60 (1.02–2.38)	163	1.62 (1.38–1.88)
Esophageal malignant neoplasm (C15)	74	1.22 (0.96–1.53)	*N* < 20	—	77	1.81 (1.43–2.26)
Gastric malignant neoplasm (C16)	262	0.92 (0.81–1.04)	37	0.64 (0.45–0.88)	299	1.09 (0.97–1.22)
Colorectal malignant neoplasms (C18–C20)	1032	1.21 (1.14–1.28)	205	1.19 (1.03–1.37)	1237	1.49 (1.40–1.57)
Liver and intrahepatic bile duct malignant neoplasm (C22)	126	1.19 (0.99–1.41)	*N* < 20	—	139	1.45 (1.22–1.72)
Gallbladder and biliary tract malignant neoplasm (C23, C24)	83	1.03 (0.82–1.28)	25	0.56 (0.36–0.83)	108	0.78 (0.64–0.94)
Pancreatic malignant neoplasm (C25)	207	1.14 (0.99–1.31)	76	1.51 (1.19–1.90)	283	1.37 (1.22–1.54)
Laryngeal malignant neoplasm (C32)	107	1.54 (1.26–1.86)	*N* < 20	—	111	2.55 (2.09–3.07)
Tracheal, bronchial, and lung malignant neoplasms (C33, C34)	1668	1.71 (1.63–1.80)	251	3.47 (3.06–3.93)	1919	2.77 (2.65–2.90)
Malignant melanoma of skin (C43)	134	1.10 (0.92–1.31)	42	1.57 (1.13–2.13)	176	1.42 (1.22–1.65)
Non‐melanoma skin malignant neoplasm (C44)	2394	1.37 (1.32–1.43)	660	1.67 (1.55–1.80)	3054	1.73 (1.67–1.80)
Connective and soft tissue malignant neoplasm, and peripheral nerves (C47, C49)	35	1.77 (1.23–2.46)	*N* < 20	—	48	2.27 (1.67–3.01)
Breast malignant neoplasm (C50) in women	—	—	287	1.33 (1.18–1.50)	287	1.33 (1.18–1.50)
Cervical malignant neoplasm (C53)	—	—	36	1.28 (0.90–1.77)	36	1.28 (0.90–1.77)
Uterine malignant neoplasm (C54, C55)	—	—	92	1.21 (0.98–1.48)	92	1.21 (0.98–1.48)
Ovarian malignant neoplasm (C56)	—	—	46	1.06 (0.77–1.41)	46	1.06 (0.77–1.41)
Prostate malignant neoplasm (C61)	1374	1.51 (1.44–1.60)	—	—	1374	1.51 (1.44–1.60)
Testicular malignant neoplasm (C62)	*N* < 20	—	—	—	*N* < 20	—
Renal malignant neoplasm (C64)	379	1.56 (1.40–1.72)	80	1.89 (1.50–2.35)	459	2.02 (1.84–2.22)
Brain, spinal cord, and other CNS parts malignant neoplasms (C70–C72)	51	1.16 (0.86–1.52)	*N* < 20	—	64	1.28 (0.99–1.64)
Thyroid gland malignant neoplasm (C73)	25	1.49 (0.96–2.20)	25	1.92 (1.24–2.84)	50	1.31 (0.97–1.73)
Hodgkin's lymphoma (HL)	*N* < 20	—	*N* < 20	—	*N* < 20	—
Non‐Hodgkin lymphoma (NHL)	122	1.28 (1.06–1.53)	46	1.80 (1.31–2.39)	168	1.56 (1.34–1.82)
Multiple myeloma and plasma cell neoplasms (MM)	47	1.01 (0.74–1.34)	*N* < 20	—	58	1.10 (0.83–1.42)
Chronic lymphocytic leukemia (CLL)	83	1.19 (0.95–1.47)	*N* < 20	—	96	1.48 (1.20–1.81)
Chronic myeloid leukemia (CML)	*N* < 20	—	*N* < 20	—	*N* < 20	—
Acute myeloid leukemia (AML)	36	1.48 (1.04–2.05)	*N* < 20	—	45	1.66 (1.21–2.22)
Acute lymphoblastic leukemia (ALL)	*N* < 20	—	*N* < 20	—	*N* < 20	—
Myelodysplastic syndromes (MDS)	36	1.92 (1.35–2.66)	*N* < 20	—	44	2.16 (1.57–2.90)
Polycythemia vera (PV)	*N* < 20	—	*N* < 20	—	*N* < 20	—
Other malignant hematologic	27	1.10 (0.72–1.60)	*N* < 20	—	30	1.19 (0.80–1.70)
Other dysplastic hematologic	*N* < 20	—	*N* < 20	—	24	1.65 (1.06–2.45)
Other malignant tumors	652	2.50 (2.31–2.70)	280	3.24 (2.87–3.64)	932	2.86 (2.68–3.05)
In situ tumors (D00–D09)	708	3.79 (3.51–4.08)	216	4.08 (3.56–4.67)	924	4.34 (4.06–4.62)
Benign neoplasms and neoplasms of unknown behavior (D10–D36, D37–D48)	197	2.79 (2.41–3.21)	67	4.16 (3.22–5.28)	264	3.62 (3.19–4.08)
Hematologic malignancy	396	1.28 (1.16–1.42)	98	1.28 (1.04–1.56)	494	1.49 (1.36–1.62)
Malignancy (C00–C97) except C44	6695	1.41 (1.37–1.44)	1635	1.57 (1.49–1.64)	8330	1.75 (1.72–1.78)
Malignancy (C00–C97)	9089	1.42 (1.39–1.45)	2295	1.62 (1.55–1.69)	11,384	1.72 (1.68–1.75)
Any neoplasia (C00–C97, D00–D09, D10–D36, D37–D48)	10,052	1.44 (1.41–1.47)	2593	1.67 (1.60–1.73)	12,645	1.78 (1.75–1.81)

*Note:* SIR calculated only for neoplasms with ≥ 20 reported cases.

In general, the risk of SPC development was the highest within the first 5 years of diagnosis of BC—53.8% (7786 cases) were diagnosed during this period, where 24.5% (3592 cases) occurred between 5 and 10 years, and 21.8% (3191 cases) after 10 years of diagnosis respectively (Table 3). The latency of SPCs relative to the diagnosis of BC is shown in Table S2.

**TABLE 3:** Risk of death due to metachronous second primary cancer in bladder cancer survivors compared to the general population (*N* = 4397).

Second primary neoplasm	Men		Women		All	
	*N*	SMR (95% IS)	*N*	SMR (95% IS)	*N*	SMR (95% IS)
Malignant neoplasms of oral cavity and pharynx (C00–C14)	55	0.73 (0.55–0.95)	*N* < 20	—	65	1.10 (0.85–1.40)
Esophageal malignant neoplasm (C15)	60	1.14 (0.87–1.46)	*N* < 20	—	63	1.71 (1.32–2.19)
Gastric malignant neoplasm (C16)	186	0.75 (0.65–0.87)	29	0.58 (0.39–0.83)	215	0.91 (0.79–1.04)
Colorectal malignant neoplasms (C18–C20)	515	0.89 (0.81–0.97)	95	0.80 (0.65–0.98)	610	1.09 (1.00–1.18)
Liver and intrahepatic bile duct malignant neoplasm (C22)	90	0.99 (0.79–1.21)	*N* < 20	—	100	1.21 (0.98–1.47)
Gallbladder and biliary tract malignant neoplasm (C23, C24)	63	0.91 (0.70–1.16)	*N* < 20	—	82	0.67 (0.53–0.83)
Pancreatic malignant neoplasm (C25)	173	1.05 (0.90–1.21)	60	1.30 (0.99–1.68)	233	1.24 (1.08–1.41)
Laryngeal malignant neoplasm (C32)	44	0.93 (0.67–1.25)	*N* < 20	—	45	1.58 (1.15–2.11)
Tracheal, bronchial, and lung malignant neoplasms (C33, C34)	1328	1.47 (1.39–1.55)	180	2.90 (2.49–3.35)	1508	2.40 (2.28–2.53)
Malignant melanoma of skin (C43)	23	0.51 (0.32–0.76)	*N* < 20	—	34	0.79 (0.54–1.10)
Non‐melanoma skin malignant neoplasm (C44)	44	0.42 (0.31–0.57)	*N* < 20	—	49	0.52 (0.38–0.69)
Connective and soft tissue malignant neoplasm, and peripheral nerves (C47, C49)	*N* < 20	—	*N* < 20	—	20	1.61 (0.98–2.48)
Breast malignant neoplasm (C50) in women	—	—	92	0.82 (0.66–1.01)	92	0.82 (0.66–1.01)
Cervical malignant neoplasm (C53)	—	—	*N* < 20	—	*N* < 20	—
Uterine malignant neoplasm (C54, C55)	—	—	21	0.62 (0.38–0.94)	21	0.62 (0.38–0.94)
Ovarian malignant neoplasm (C56)	—	—	31	0.88 (0.60–1.25)	31	0.88 (0.60–1.25)
Prostate malignant neoplasm (C61)	323	0.82 (0.73–0.92)	—	—	323	0.82 (0.73–0.92)
Testicular malignant neoplasm (C62)	*N* < 20	—	—	—	*N* < 20	—
Renal malignant neoplasm (C64)	146	1.06 (0.90–1.25)	35	1.50 (1.04–2.08)	181	1.46 (1.26–1.69)
Brain, spinal cord, and other CNS parts malignant neoplasms (C70–C72)	40	1.02 (0.73–1.39)	*N* < 20	—	48	1.09 (0.80–1.44)
Thyroid gland malignant neoplasm (C73)	*N* < 20	—	*N* < 20	—	*N* < 20	—
Hodgkin's lymphoma (HL)	*N* < 20	—	*N* < 20	—	*N* < 20	—
Non‐Hodgkin lymphoma (NHL)	60	0.95 (0.73–1.23)	20	1.23 (0.75–1.91)	80	1.18 (0.93–1.46)
Multiple myeloma and plasma cell neoplasms (MM)	28	0.81 (0.54–1.17)	*N* < 20	—	37	0.94 (0.66–1.29)
Chronic lymphocytic leukemia (CLL)	25	0.58 (0.38–0.86)	*N* < 20	—	31	0.81 (0.55–1.15)
Chronic myeloid leukemia (CML)	*N* < 20	—	*N* < 20	—	*N* < 20	—
Acute myeloid leukemia (AML)	26	1.21 (0.79–1.77)	*N* < 20	—	31	1.29 (0.88–1.83)
Acute lymphoblastic leukemia (ALL)	*N* < 20	—	*N* < 20	—	*N* < 20	—
Myelodysplastic syndromes (MDS)	*N* < 20	—	*N* < 20	—	*N* < 20	—
Polycythemia vera (PV)	*N* < 20	—	*N* < 20	—	*N* < 20	—
Other malignant hematologic	*N* < 20	—	*N* < 20	—	*N* < 20	—
Other dysplastic hematologic	*N* < 20	—	*N* < 20	—	*N* < 20	—
Other malignant tumors	272	1.41 (1.25–1.59)	118	1.79 (1.48–2.14)	390	1.60 (1.45–1.77)
In situ tumors (D00–D09)	*N* < 20	—	*N* < 20	—	*N* < 20	—
Benign neoplasms and neoplasms of unknown behavior (D10–D36, D37–D48)	*N* < 20	—	*N* < 20	—	*N* < 20	—
Hematologic malignancy	185	0.86 (0.74–0.99)	50	0.95 (0.70–1.25)	235	1.04 (0.91–1.18)
Malignancy (C00–C97) except C44	3508	1.02 (0.99–1.06)	794	1.10 (1.02–1.17)	4302	1.25 (1.21–1.29)
Malignancy (C00–C97)	3552	1.01 (0.97–1.04)	799	1.07 (1.00–1.15)	4351	1.27 (1.23–1.31)
Any neoplasia (C00–C97, D00–D09, D10–D36, D37–D48)	3579	1.01 (0.97–1.04)	818	1.08 (1.01–1.16)	4397	1.25 (1.22–1.29)

*Note:* SMR calculated only for neoplasms with ≥ 20 reported deaths.

BC survivors with stage I BC at diagnosis were the most numerous subgroup of patients with SPCs (7523 SPC cases, 50.3%). Notably, potentially carcinogenic chemotherapy and radiation therapy are usually not used in these patients. As shown in Table S3, the risk of SPC remained stable during follow‐up for stage I BC, except for prostate cancer which tended to be diagnosed early after stage I BC diagnosis, and non‐melanoma skin tumors and pancreatic cancer, which were diagnosed late after stage I BC.

### 3.3 The Relative Risk of Death due to Second Primary Cancers Following Bladder Cancer

**FIGURE 2:**
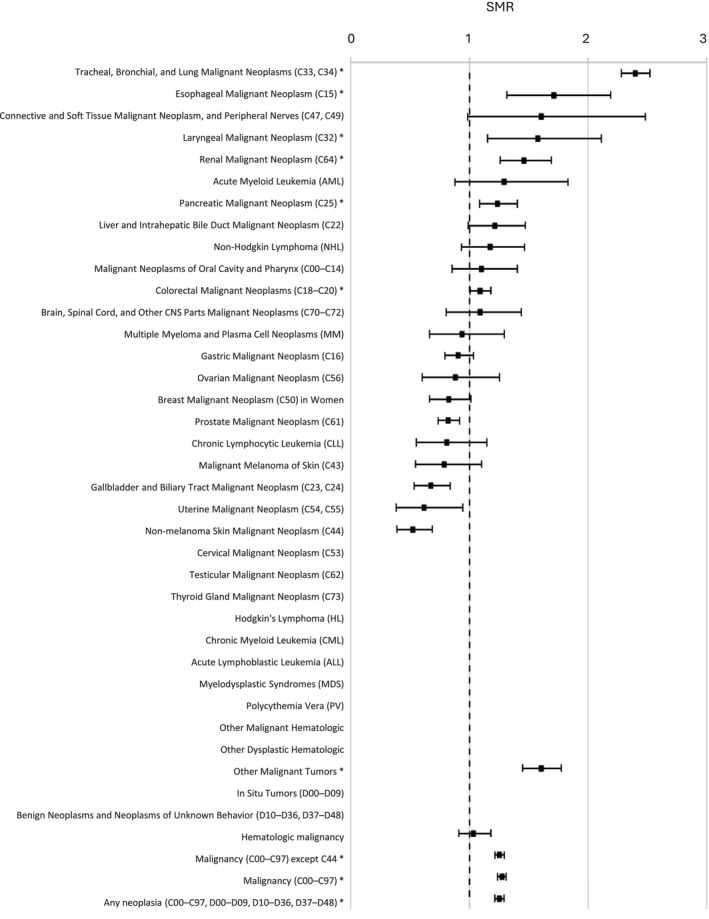
Risk of death due to second primary cancer in bladder cancer survivors compared to the general population.

The SMR was increased in BC survivors (1.25; 95% CI 1.21–1.29) regarding all‐cancer mortality (excluding non‐melanoma skin cancers). There was a greatly increased risk of death caused by lung cancer in the population of BC survivors, with the SMR of 2.40 (95% CI 2.28–2.53), especially in women with an SMR of 2.90 (2.49–3.35). There was also an increased mortality due to esophageal, colorectal, pancreatic, and renal cancer, but lower mortality associated with biliary cancer, non‐melanoma skin cancer, and uterine neoplasms (Table 3; Figure 2).

### 3.4 Clinical Stage of Second Primary Cancers at Diagnosis

For selected types of SPCs, clinical stage at diagnosis was compared to that in the general population with data in the National Cancer Registry (*n* = 1,163,537) (Table 4). Colorectal cancer and non‐melanoma skin tumors were diagnosed at a less advanced stage in BC survivors regardless of the time from BC diagnosis. For prostate cancer, BC survivors had a higher probability of being diagnosed at stages I or II. Similarly, BC survivors with renal cancer were also found to be more frequently diagnosed at an earlier stage compared to reference clinical stage distribution. In contrast, lung cancer was more likely to be metastatic at diagnosis in BC survivors (Table 4).

**TABLE 4:** Clinical stage of selected types of metachronous second primary cancers (SPCs) according to time since bladder cancer (BC) diagnosis (*N* = 9690) compared to stage distribution for first cancers in the Czech National Registry (*N* = 1,163,537).

Second primary neoplasm	SPC < 5 years after BC	SPC 5–10 years after BC	SPC > 10 years after BC	First cancer (*N* = 1,163,537)	Comparison of stage distribution (Fisher's exact test)		
					SPC < 5 years after BC	SPC 5–10 years after BC	SPC > 10 years after BC
Colorectal malignant neoplasms (C18–C20)							
I	178 (23.2%)	76 (23.9%)	79 (23.4%)	49,133 (20.9%)	*p* < 0.001	*p* = 0.001	*p* = 0.001
II	190 (24.8%)	78 (24.5%)	87 (25.7%)	46,766 (19.9%)			
III	140 (18.3%)	66 (20.8%)	68 (20.1%)	41,103 (17.5%)			
IV	142 (18.5%)	59 (18.6%)	60 (17.8%)	50,085 (21.3%)			
Not reported	117 (15.3%)	39 (12.3%)	44 (13.0%)	48,267 (20.5%)			
Tracheal, bronchial, and lung malignant neoplasms (C33, C34)							
I	165 (15.0%)	61 (10.9%)	49 (10.8%)	27,089 (12.1%)	*p* = 0.017	*p* = 0.117	*p* = 0.018
II	117 (10.7%)	46 (8.2%)	40 (8.8%)	24,228 (10.8%)			
III	244 (22.2%)	147 (26.3%)	97 (21.5%)	56,696 (25.3%)			
IV	364 (33.2%)	205 (36.7%)	180 (39.8%)	73,200 (32.7%)			
Not reported	207 (18.9%)	100 (17.9%)	86 (19.0%)	42,569 (19.0%)			
Non‐melanoma skin malignant neoplasm (C44)							
I	1282 (87.3%)	794 (89.2%)	816 (89.3%)	274,955 (83.3%)	*p* < 0.001	*p* < 0.001	*p* < 0.001
II	104 (7.1%)	65 (7.3%)	73 (8.0%)	23,951 (7.3%)			
III	10 (0.7%)	4 (0.4%)	4 (0.4%)	1469 (0.4%)			
IV	1 (0.1%)	1 (0.1%)	0 (0.0%)	531 (0.2%)			
Not reported	71 (4.8%)	26 (2.9%)	21 (2.3%)	29,361 (8.9%)			
Breast malignant neoplasm (C50) in women							
I	48 (31.0%)	34 (36.2%)	25 (35.7%)	51,878 (29.3%)	*p* = 0.074	*p* = 0.325	*p* = 0.110
II	52 (33.5%)	30 (31.9%)	28 (40.0%)	66,331 (37.5%)			
III	20 (12.9%)	13 (13.8%)	5 (7.1%)	30,699 (17.3%)			
IV	16 (10.3%)	7 (7.4%)	9 (12.9%)	15,337 (8.7%)			
Not reported	19 (12.3%)	10 (10.6%)	3 (4.3%)	12,748 (7.2%)			
Prostate malignant neoplasm (C61)							
I	344 (27.3%)	101 (25.5%)	87 (30.6%)	23,861 (19.2%)	*p* < 0.001	*p* = 0.002	*p* < 0.001
II	446 (35.3%)	136 (34.3%)	108 (38.0%)	38,600 (31.0%)			
III	93 (7.4%)	28 (7.1%)	19 (6.7%)	11,884 (9.6%)			
IV	131 (10.4%)	51 (12.9%)	32 (11.3%)	20,180 (16.2%)			
Not reported	248 (19.7%)	80 (20.2%)	38 (13.4%)	29,841 (24.0%)			
Renal malignant neoplasm (C64)							
I	164 (44.1%)	52 (40.0%)	54 (43.5%)	19,396 (26.7%)	*p* < 0.001	*p* = 0.004	*p* = 0.001
II	35 (9.4%)	14 (10.8%)	15 (12.1%)	11,417 (15.7%)			
III	33 (8.9%)	11 (8.5%)	13 (10.5%)	8681 (11.9%)			
IV	52 (14.0%)	18 (13.8%)	23 (18.5%)	15,344 (21.1%)			
Not reported	88 (23.7%)	35 (26.9%)	19 (15.3%)	17,937 (24.6%)			

## 4 Discussion

Second primary cancer is a serious complication significantly affecting overall survival and quality of life in cancer patients. Various factors can explain the increased risk of malignancies in BC survivors. Cigarette smoking is the most important risk factor for BC and many other malignancies highlighted in the present analysis. The increased incidence of prostate and renal cancer as a SPC in the first year after BC diagnosis can be explained by detecting incidental, asymptomatic cancers during urology investigations carried out as a part of BC staging and treatment. Interestingly, there was lower mortality in BC survivors compared to the general population of biliary cancer, non‐melanoma skin cancer, and uterine neoplasms, possibly pointing to earlier diagnosis during investigations and follow‐up for BC.

Nevertheless, there were some malignancies where the association cannot be explained by a common risk factor. Women after BC had a higher risk of thyroid gland cancer. There was an increased risk of both melanomas and non‐melanoma skin cancers, and of chronic lymphocytic leukemia. The SIR for central nervous system malignancies was bordering on clinical significance. Remarkably, soft tissue tumors had a SIR of 2.27 (95% CI 2.09–3.07), although the number of cases was relatively low (Table S1). These malignancies may be associated with the use of diagnostic ionizing radiation and cytotoxic chemotherapy or radiation therapy for BC.

Some of the differences between the populations of BC patients who did develop SPC versus those that did not can be explained by the immortality bias: patients with SPC were younger at the diagnosis of BC, and were diagnosed with a less advanced clinical stage. Competing causes of mortality in the elderly population limit the relative risk of second cancers.

The problem of SPCs after BC has been the subject of several previously published studies.

Zecha et al. in their study published in 2011 focused on non‐muscle‐invasive BC patients, revealing a significant incidence of second malignancies, especially prostate and lung cancers in men, and lung and uterine cancers in women [5]. A study based on the Netherlands Cancer Registry also analyzed the impact of treatment data and found an elevated risk of SPCs in BC patients, especially among younger individuals and those treated with radiotherapy or chemotherapy [6].

Risk factors for the development of SPCs were addressed by Shiels and collaborators who investigated the association between pre‐diagnostic smoking and the risk of second smoking‐associated cancers. They found significantly increased risks for survivors of stage I lung, bladder, head/neck, and kidney cancers who were current heavy smokers [7]. The burden of smoking‐related SPC has also been the subject of another large US‐based study, finding a high risk in survivors with specific site‐associated cancers like bladder and head and neck cancers. This study highlighted the declining smoking prevalence but pointed to the continuing risk of SPCs in survivors of smoking‐related cancers [3]. Khanal et al. (2017) utilized data from the SEER 18 database and reported that 7.5% of patients with primary malignancies at smoking‐related cancer sites developed SPCs, with survivors of head and neck cancer and BC experiencing the highest risk [4].

A population‐based cohort study in Korea analyzed data from 48,875 individuals diagnosed with BC. Their findings surprisingly indicated a 6% lower overall risk of SPCs compared to the general population, but a higher risk for specific cancers such as prostate and lung cancers [11]. In contrast, Lehnert et al. (2012) evaluated new malignancies following BC in Germany. They reported elevated risks for secondary cancer, particularly respiratory tract and prostate cancers [8]. Similarly, Muller et al. reported a 60% higher risk of new malignancies among BC survivors in France, with increases in lung, head and neck, and prostate cancers [10]. In a study by Şahin et al. 2339 patients with urothelial cancer were analyzed. Of these patients, 11.1% developed an SPC, predominantly lung cancer [9]. Finally a large Surveillance, Epidemiology, and End Results (SEER)‐based mortality analysis of patients with non‐muscle‐invasive bladder cancer was recently published by Slusarczyk and collaborators. The major causes of cancer death included BC, lung cancer, prostate cancer, hematological malignancies, and upper urinary tract urothelial cancer [16]. Our findings are consistent with registry‐based analyses from the United States (SEER) and other countries. Although the methodology varies among these published reports, the results reported by Khanal et al. in 2017 based on the SEER‐based analysis of more than 100,000 BC cases, reported similar SIRs for smoking‐related and selected non‐smoking‐related cancers, in particular prostate cancer, among BC survivors [4]. However, SEER data in contrast to the Czech registry do not include information on SPC stage [4].

These studies collectively underscore the heightened risk of SPCs in BC survivors, the influence of shared etiological factors such as smoking, indicating the possibility and the importance of targeted surveillance and smoking cessation interventions in managing the risks. The risk most lethal and frequent of these SPCs, lung cancer, remains stable over a long‐term follow‐up period.

The strengths of this study include a large cohort and a long‐term follow‐up achieved thanks to the continuum of the CNCR data. The limitations include the lack of information about socio‐demographic status, ethnicity, comorbidities, risk factors, and therapy regimens. CNCR (especially pre‐1990 entries) also includes a significant proportion of cancer patients missing tumor type and tumor stage data. We decided not to include the patients with SPCs detected within the first 6 months of the diagnosis of BC because they represent a different medical scenario. These are often incidental cancers that are detected during staging investigations for the first cancer, and treatment and other medical interventions administered for the first cancer do not play a role in their etiology. However, the used interval of 6 months to differentiate between synchronous and metachronous cancer is arbitrary.

Urothelial SPCs were not included in our analysis for several reasons. Field changes of the urinary tract mucosa underlie the development of urothelial malignancies and second cancers may be indistinguishable from local recurrences and seeding of the first cancer in the urothelial tract [17, 18]. Furthermore, it is frequently impossible to reliably ascertain which of the tumors produces metastases and eventually leads to death, confounding SMR calculations.

In conclusion, the present registry‐based study, one of the largest and most detailed published to date, shows that BC survivors have a significantly higher risk of being diagnosed with SPC than the general population, especially during the first 5 years after BC presentation. A personalized follow‐up and surveillance strategy is needed to decrease SPC‐associated morbidity and mortality.

## Author Contributions

**Tomas Buchler:** conceptualization (equal), data curation (equal), investigation (equal), methodology (equal), project administration (equal), supervision (equal), writing – original draft (equal), writing – review and editing (equal). **Lucie Pehalova Kusova:** data curation (equal), formal analysis (equal), investigation (equal), methodology (equal), validation (equal), writing – review and editing (equal). **Alina Pirshtuk:** data curation (equal), writing – original draft (supporting), writing – review and editing (equal). **Jan Muzik:** conceptualization (equal), data curation (equal), formal analysis (equal), writing – review and editing (equal). **Marek Babjuk:** data curation (equal), validation (equal), writing – review and editing (equal). **Ladislav Dusek:** formal analysis (equal), resources (equal), supervision (equal), validation (equal), writing – review and editing (equal).

## Funding

The authors have nothing to report.

## Ethics Statement

This study used anonymised, aggregated data from the Czech National Cancer Registry. According to Czech national regulations (Act No. 372/2011 Coll., on Health Services) and institutional policy, analyses of de‐identified registry data are exempt from ethical review. The study was conducted in accordance with the Declaration of Helsinki and its amendments.

## Conflicts of Interest

Tomas Buchler has received research support and honoraria from Roche, Bristol Myers Squibb, Merck Sharp Dohme, Merck, Ipsen, Novartis, and AstraZeneca, all unrelated to the present paper. Other authors declare no conflicts of interest.

## Novelty and Impact Statement

The understanding of the risk of specific second primary cancers (SPCs) is important to improve risk management strategy for bladder cancer (BC) survivors. The aim of this registry‐based study was to investigate the risk of SPC in BC survivors using extensive, long‐term and validated information from the Czech National Cancer Registry. Data of 12 645 patients with a SPC was analyzed, one of the largest cohorts published to date.

## Supporting information


**Table S1:** Neoplasms diagnosed after bladder cancer (*N* = 14,669).
**Table S2:** Interval from bladder cancer diagnosis to second primary cancer diagnosis (*N* = 12,645).
**Table S3:** Neoplasms diagnosed after stage I bladder cancer (*N* = 7523).

## Data Availability

The data that support the findings of this study are available on request from the corresponding author. The data are not publicly available due to privacy or ethical restrictions.
