# Supplementary material for: Second Primary Cancer After Bladder Cancer: A Comprehensive Analysis of a National Cancer Registry
Source: Cancer Med. 2025 Nov 30;14(23):e71427. doi: 10.1002/cam4.71427 (PMC12665869; doi:10.1002/cam4.71427)
Supplement: Supplementary file 1 — Table S1: Neoplasms diagnosed after bladder cancer (N = 14,669). Table S2: Interval from bladder cancer diagnosis to second primary cancer diagnosis (N = 12,645). Table S3: Neoplasms diagnosed after stage I bladder cancer (N = 7523). [file CAM4-14-e71427-s001.docx]

**Supplementary Table S1.** Neoplasms diagnosed after bladder cancer (N = 14 669).

| **Second primary neoplasm** | **Within 6 months after BC (synchronous)**  **(N = 2 024)** | **After 6 months after BC (metachronous)**  **(N = 12 645)** | **All**  **(N = 14 669)** |
| --- | --- | --- | --- |
| Malignant Neoplasms of Oral Cavity and Pharynx (C00–C14) | 15 (0.7 %) | 163 (1.3 %) | 178 (1.2 %) |
| Esophageal Malignant Neoplasm (C15) | 8 (0.4 %) | 77 (0.6 %) | 85 (0.6 %) |
| Gastric Malignant Neoplasm (C16) | 31 (1.5 %) | 299 (2.4 %) | 330 (2.2 %) |
| Colorectal Malignant Neoplasms (C18–C20) | 186 (9.2 %) | 1 237 (9.8 %) | 1 423 (9.7 %) |
| Liver and Intrahepatic Bile Duct Malignant Neoplasm (C22) | 16 (0.8 %) | 139 (1.1 %) | 155 (1.1 %) |
| Gallbladder and Biliary Tract Malignant Neoplasm (C23, C24) | 10 (0.5 %) | 108 (0.9 %) | 118 (0.8 %) |
| Pancreatic Malignant Neoplasm (C25) | 22 (1.1 %) | 283 (2.2 %) | 305 (2.1 %) |
| Laryngeal Malignant Neoplasm (C32) | 6 (0.3 %) | 111 (0.9 %) | 117 (0.8 %) |
| Tracheal, Bronchial, and Lung Malignant Neoplasms (C33, C34) | 189 (9.3 %) | 1 919 (15.2 %) | 2 108 (14.4 %) |
| Malignant Melanoma of Skin (C43) | 14 (0.7 %) | 176 (1.4 %) | 190 (1.3 %) |
| Non-melanoma Skin Malignant Neoplasm (C44) | 218 (10.8 %) | 3 054 (24.2 %) | 3 272 (22.3 %) |
| Connective and Soft Tissue Malignant Neoplasm, and Peripheral Nerves (C47, C49) | 5 (0.2 %) | 48 (0.4 %) | 53 (0.4 %) |
| Breast Malignant Neoplasm (C50) in Women | 32 (1.6 %) | 287 (2.3 %) | 319 (2.2 %) |
| Cervical Malignant Neoplasm (C53) | 24 (1.2 %) | 36 (0.3 %) | 60 (0.4 %) |
| Uterine Malignant Neoplasm (C54, C55) | 12 (0.6 %) | 92 (0.7 %) | 104 (0.7 %) |
| Ovarian Malignant Neoplasm (C56) | 11 (0.5 %) | 46 (0.4 %) | 57 (0.4 %) |
| Prostate Malignant Neoplasm (C61) | 568 (28.1 %) | 1 374 (10.9 %) | 1 942 (13.2 %) |
| Testicular Malignant Neoplasm (C62) | 0 (0.0 %) | 9 (0.1 %) | 9 (0.1 %) |
| Renal Malignant Neoplasm (C64) | 167 (8.3 %) | 459 (3.6 %) | 626 (4.3 %) |
| Brain, Spinal Cord, and Other CNS Parts Malignant Neoplasms (C70–C72) | 6 (0.3 %) | 64 (0.5 %) | 70 (0.5 %) |
| Thyroid Gland Malignant Neoplasm (C73) | 3 (0.1 %) | 50 (0.4 %) | 53 (0.4 %) |
| Hodgkin's Lymphoma (HL) | 2 (0.1 %) | 12 (0.1 %) | 14 (0.1 %) |
| Non-Hodgkin Lymphoma (NHL) | 28 (1.4 %) | 168 (1.3 %) | 196 (1.3 %) |
| Multiple Myeloma and Plasma Cell Neoplasms (MM) | 9 (0.4 %) | 58 (0.5 %) | 67 (0.5 %) |
| Chronic Lymphocytic Leukemia (CLL) | 18 (0.9 %) | 96 (0.8 %) | 114 (0.8 %) |
| Chronic Myeloid Leukemia (CML) | 1 (0.0 %) | 7 (0.1 %) | 8 (0.1 %) |
| Acute Myeloid Leukemia (AML) | 6 (0.3 %) | 45 (0.4 %) | 51 (0.3 %) |
| Acute Lymphoblastic Leukemia (ALL) | 1 (0.0 %) | 5 (0.0 %) | 6 (0.0 %) |
| Myelodysplastic Syndromes (MDS) | 3 (0.1 %) | 44 (0.3 %) | 47 (0.3 %) |
| Polycythemia Vera (PV) | 2 (0.1 %) | 5 (0.0 %) | 7 (0.0 %) |
| Other Malignant Hematologic | 0 (0.0 %) | 30 (0.2 %) | 30 (0.2 %) |
| Other Dysplastic Hematologic | 3 (0.1 %) | 24 (0.2 %) | 27 (0.2 %) |
| Other Malignant Tumors | 223 (11.0 %) | 932 (7.4 %) | 1 155 (7.9 %) |
| In Situ Tumors (D00–D09) | 140 (6.9 %) | 924 (7.3 %) | 1 064 (7.3 %) |
| Benign Neoplasms and Neoplasms of Unknown Behavior (D10–D36, D37–D48) | 45 (2.2 %) | 264 (2.1 %) | 309 (2.1 %) |

**Supplementary Table S2.** Interval from bladder cancer diagnosis to second primary cancer diagnosis (N = 12 645).

| **Second primary neoplasm** | **Men** | | | **Women** | | | | | | **All** | | | | | |
| --- | --- | --- | --- | --- | --- | --- | --- | --- | --- | --- | --- | --- | --- | --- | --- |
|  | **N** | **Interval (years)** | | **N** | **Interval (years)** | | | | **N** | | **Interval (years)** | | | |  |
|  |  | **Mean** | **Median** |  | **Mean** | | **Median** | |  |  | **Mean** | | **Median** | |  |
| Malignant Neoplasms of Oral Cavity and Pharynx (C00–C14) | 139 | 4.9 | 6.8 | 24 | | 5.7 | | 7.0 | | 163 | | 5.3 | | 6.8 | |
| Esophageal Malignant Neoplasm (C15) | 74 | 4.4 | 6.3 | N < 20 | | – | | – | | 77 | | 4.5 | | 6.4 | |
| Gastric Malignant Neoplasm (C16) | 262 | 4.5 | 6.5 | 37 | | 5.4 | | 7.2 | | 299 | | 4.7 | | 6.6 | |
| Colorectal Malignant Neoplasms (C18–C20) | 1 032 | 5.2 | 7.1 | 205 | | 5.8 | | 8.1 | | 1 237 | | 5.3 | | 7.3 | |
| Liver and Intrahepatic Bile Duct Malignant Neoplasm (C22) | 126 | 5.7 | 6.9 | N < 20 | | – | | – | | 139 | | 5.5 | | 6.8 | |
| Gallbladder and Biliary Tract Malignant Neoplasm (C23, C24) | 83 | 5.2 | 6.2 | 25 | | 6.0 | | 7.7 | | 108 | | 5.3 | | 6.5 | |
| Pancreatic Malignant Neoplasm (C25) | 207 | 6.0 | 7.3 | 76 | | 6.6 | | 7.9 | | 283 | | 6.3 | | 7.5 | |
| Laryngeal Malignant Neoplasm (C32) | 107 | 5.0 | 6.6 | N < 20 | | – | | – | | 111 | | 4.9 | | 6.6 | |
| Tracheal, Bronchial, and Lung Malignant Neoplasms (C33, C34) | 1 668 | 5.2 | 6.7 | 251 | | 6.7 | | 7.6 | | 1 919 | | 5.3 | | 6.8 | |
| Malignant Melanoma of Skin (C43) | 134 | 6.4 | 7.8 | 42 | | 5.8 | | 6.6 | | 176 | | 6.4 | | 7.5 | |
| Non-melanoma Skin Malignant Neoplasm (C44) | 2 394 | 6.3 | 7.8 | 660 | | 7.1 | | 8.4 | | 3 054 | | 6.4 | | 7.9 | |
| Connective and Soft Tissue Malignant Neoplasm, and Peripheral Nerves (C47, C49) | 35 | 4.9 | 6.5 | N < 20 | | – | | – | | 48 | | 5.2 | | 7.2 | |
| Breast Malignant Neoplasm (C50) in Women | – | – | – | 287 | | 6.1 | | 7.2 | | 287 | | 6.1 | | 7.2 | |
| Cervical Malignant Neoplasm (C53) | – | – | – | 36 | | 4.2 | | 6.1 | | 36 | | 4.2 | | 6.1 | |
| Uterine Malignant Neoplasm (C54, C55) | – | – | – | 92 | | 5.2 | | 7.0 | | 92 | | 5.2 | | 7.0 | |
| Ovarian Malignant Neoplasm (C56) | – | – | – | 46 | | 5.3 | | 6.7 | | 46 | | 5.3 | | 6.7 | |
| Prostate Malignant Neoplasm (C61) | 1 374 | 4.9 | 6.2 | – | | – | | – | | 1 374 | | 4.9 | | 6.2 | |
| Testicular Malignant Neoplasm (C62) | N < 20 | – | – | – | | – | | – | | N < 20 | | – | | – | |
| Renal Malignant Neoplasm (C64) | 379 | 6.0 | 7.5 | 80 | | 3.7 | | 6.2 | | 459 | | 5.6 | | 7.3 | |
| Brain, Spinal Cord, and Other CNS Parts Malignant Neoplasms (C70–C72) | 51 | 4.7 | 7.5 | N < 20 | | – | | – | | 64 | | 4.6 | | 7.1 | |
| Thyroid Gland Malignant Neoplasm (C73) | 25 | 9.3 | 10.0 | 25 | | 7.1 | | 7.6 | | 50 | | 7.1 | | 8.8 | |
| Hodgkin's Lymphoma (HL) | N < 20 | – | – | N < 20 | | – | | – | | N < 20 | | – | | – | |
| Non-Hodgkin Lymphoma (NHL) | 122 | 4.8 | 7.3 | 46 | | 5.8 | | 6.7 | | 168 | | 5.4 | | 7.1 | |
| Multiple Myeloma and Plasma Cell Neoplasms (MM) | 47 | 5.0 | 6.6 | N < 20 | | – | | – | | 58 | | 4.8 | | 6.6 | |
| Chronic Lymphocytic Leukemia (CLL) | 83 | 6.6 | 7.6 | N < 20 | | – | | – | | 96 | | 6.7 | | 7.6 | |
| Chronic Myeloid Leukemia (CML) | N < 20 | – | – | N < 20 | | – | | – | | N < 20 | | – | | – | |
| Acute Myeloid Leukemia (AML) | 36 | 5.3 | 6.6 | N < 20 | | – | | – | | 45 | | 4.9 | | 6.3 | |
| Acute Lymphoblastic Leukemia (ALL) | N < 20 | – | – | N < 20 | | – | | – | | N < 20 | | – | | – | |
| Myelodysplastic Syndromes (MDS) | 36 | 7.1 | 8.7 | N < 20 | | – | | – | | 44 | | 6.3 | | 8.4 | |
| Polycythemia Vera (PV) | N < 20 | – | – | N < 20 | | – | | – | | N < 20 | | – | | – | |
| Other Malignant Hematologic | 27 | 6.5 | 7.2 | N < 20 | | – | | – | | 30 | | 6.5 | | 7.3 | |
| Other Dysplastic Hematologic | N < 20 | – | – | N < 20 | | – | | – | | 24 | | 5.8 | | 6.6 | |
| Other Malignant Tumors | 652 | 5.0 | 6.5 | 280 | | 4.8 | | 6.0 | | 932 | | 5.0 | | 6.4 | |
| In Situ Tumors (D00–D09) | 708 | 4.6 | 6.4 | 216 | | 4.9 | | 6.9 | | 924 | | 4.7 | | 6.5 | |
| Benign Neoplasms and Neoplasms of Unknown Behavior (D10–D36, D37–D48) | 197 | 5.5 | 7.3 | 67 | | 5.2 | | 7.2 | | 264 | | 5.4 | | 7.3 | |
| Hematologic malignancy | 396 | 5.5 | 7.3 | 98 | | 5.5 | | 6.6 | | 494 | | 5.5 | | 7.1 | |
| Malignancy (C00–C97) except C44 | 6 695 | 5.2 | 6.8 | 1 635 | | 5.7 | | 7.1 | | 8 330 | | 5.2 | | 6.8 | |
| Malignancy (C00–C97) | 9 089 | 5.4 | 7.0 | 2 295 | | 6.0 | | 7.4 | | 11 384 | | 5.5 | | 7.1 | |
| Any neoplasia (C00–C97, D00–D09, D10–D36, D37–D48) | 10 052 | 5.4 | 7.0 | 2 593 | | 5.9 | | 7.4 | | 12 645 | | 5.5 | | 7.1 | |

*Only for neoplasms with ≥ 20 cases*

**Supplementary Table S3.** Neoplasms diagnosed after stage I bladder cancer (N = 7 523).

| **Second primary neoplasm** | **SPC <5 years after BC**  **(N = 4 183)** | **SPC 5-10 years after BC**  **(N = 2 011)** | **SPC >10 years after BC**  **(N = 1 329)** |
| --- | --- | --- | --- |
| Malignant Neoplasms of Oral Cavity and Pharynx (C00–C14) | 47 (1.1 %) | 21 (1.0 %) | 18 (1.4 %) |
| Esophageal Malignant Neoplasm (C15) | 25 (0.6 %) | 9 (0.4 %) | 5 (0.4 %) |
| Gastric Malignant Neoplasm (C16) | 76 (1.8 %) | 35 (1.7 %) | 22 (1.7 %) |
| Colorectal Malignant Neoplasms (C18–C20) | 383 (9.2 %) | 158 (7.9 %) | 130 (9.8 %) |
| Liver and Intrahepatic Bile Duct Malignant Neoplasm (C22) | 38 (0.9 %) | 26 (1.3 %) | 12 (0.9 %) |
| Gallbladder and Biliary Tract Malignant Neoplasm (C23, C24) | 19 (0.5 %) | 17 (0.8 %) | 8 (0.6 %) |
| Pancreatic Malignant Neoplasm (C25) | 60 (1.4 %) | 48 (2.4 %) | 33 (2.5 %) |
| Laryngeal Malignant Neoplasm (C32) | 31 (0.7 %) | 19 (0.9 %) | 10 (0.8 %) |
| Tracheal, Bronchial, and Lung Malignant Neoplasms (C33, C34) | 523 (12.5 %) | 270 (13.4 %) | 180 (13.5 %) |
| Malignant Melanoma of Skin (C43) | 46 (1.1 %) | 37 (1.8 %) | 29 (2.2 %) |
| Non-melanoma Skin Malignant Neoplasm (C44) | 844 (20.2 %) | 518 (25.8 %) | 353 (26.6 %) |
| Connective and Soft Tissue Malignant Neoplasm, and Peripheral Nerves (C47, C49) | 14 (0.3 %) | 5 (0.2 %) | 5 (0.4 %) |
| Breast Malignant Neoplasm (C50) in Women | 80 (1.9 %) | 44 (2.2 %) | 25 (1.9 %) |
| Cervical Malignant Neoplasm (C53) | 17 (0.4 %) | 7 (0.3 %) | 2 (0.2 %) |
| Uterine Malignant Neoplasm (C54, C55) | 33 (0.8 %) | 15 (0.7 %) | 6 (0.5 %) |
| Ovarian Malignant Neoplasm (C56) | 10 (0.2 %) | 8 (0.4 %) | 5 (0.4 %) |
| Prostate Malignant Neoplasm (C61) | 559 (13.4 %) | 228 (11.3 %) | 121 (9.1 %) |
| Testicular Malignant Neoplasm (C62) | 2 (0.0 %) | 1 (0.0 %) | 1 (0.1 %) |
| Renal Malignant Neoplasm (C64) | 205 (4.9 %) | 73 (3.6 %) | 54 (4.1 %) |
| Brain, Spinal Cord, and Other CNS Parts Malignant Neoplasms (C70–C72) | 25 (0.6 %) | 7 (0.3 %) | 6 (0.5 %) |
| Thyroid Gland Malignant Neoplasm (C73) | 16 (0.4 %) | 5 (0.2 %) | 9 (0.7 %) |
| Hodgkin's Lymphoma (HL) | 4 (0.1 %) | 1 (0.0 %) | 1 (0.1 %) |
| Non-Hodgkin Lymphoma (NHL) | 61 (1.5 %) | 29 (1.4 %) | 20 (1.5 %) |
| Multiple Myeloma and Plasma Cell Neoplasms (MM) | 18 (0.4 %) | 11 (0.5 %) | 2 (0.2 %) |
| Chronic Lymphocytic Leukemia (CLL) | 28 (0.7 %) | 14 (0.7 %) | 8 (0.6 %) |
| Chronic Myeloid Leukemia (CML) | 0 (0.0 %) | 0 (0.0 %) | 0 (0.0 %) |
| Acute Myeloid Leukemia (AML) | 12 (0.3 %) | 9 (0.4 %) | 3 (0.2 %) |
| Acute Lymphoblastic Leukemia (ALL) | 4 (0.1 %) | 0 (0.0 %) | 0 (0.0 %) |
| Myelodysplastic Syndromes (MDS) | 13 (0.3 %) | 9 (0.4 %) | 6 (0.5 %) |
| Polycythemia Vera (PV) | 2 (0.0 %) | 1 (0.0 %) | 1 (0.1 %) |
| Other Malignant Hematologic | 5 (0.1 %) | 0 (0.0 %) | 5 (0.4 %) |
| Other Dysplastic Hematologic | 12 (0.3 %) | 5 (0.2 %) | 0 (0.0 %) |
| Other Malignant Tumors | 366 (8.7 %) | 152 (7.6 %) | 83 (6.2 %) |
| In Situ Tumors (D00–D09) | 486 (11.6 %) | 183 (9.1 %) | 119 (9.0 %) |
| Benign Neoplasms and Neoplasms of Unknown Behavior (D10–D36, D37–D48) | 119 (2.8 %) | 46 (2.3 %) | 47 (3.5 %) |
